# ATBF1 and NQO1 as candidate targets for allelic loss at chromosome arm 16q in breast cancer: Absence of somatic ATBF1 mutations and no role for the C609T NQO1 polymorphism

**DOI:** 10.1186/1471-2407-8-105

**Published:** 2008-04-16

**Authors:** Anne-Marie Cleton-Jansen, Ronald van Eijk, Marcel Lombaerts, Marjanka K Schmidt, Laura J Van't Veer, Katja Philippo, Rhyenne ME Zimmerman, Johannes L Peterse, Vincent TBHM Smit, Tom van Wezel, Cees J Cornelisse

**Affiliations:** 1 Department of Pathology, Leiden University Medical Centre, Leiden, The Netherlands; 2 Department of Epidemiology, Netherlands Cancer Institute-Antoni van Leeuwenhoek Hospital, Amsterdam, The Netherlands; 3 Department of Pathology, Netherlands Cancer Institute-Antoni van Leeuwenhoek Hospital, Amsterdam, The Netherlands

## Abstract

**Background:**

Loss of heterozygosity (LOH) at chromosome arm 16q is frequently observed in human breast cancer, suggesting that one or more target tumor suppressor genes (TSGs) are located there. However, detailed mapping of the smallest region of LOH has not yet resulted in the identification of a TSG at 16q. Therefore, the present study attempted to identify TSGs using an approach based on mRNA expression.

**Methods:**

A cDNA microarray for the 16q region was constructed and analyzed using RNA samples from 39 breast tumors with known LOH status at 16q.

**Results:**

Five genes were identified to show lower expression in tumors with LOH at 16q compared to tumors without LOH. The genes for NAD(P)H dehydrogenase quinone (*NQO1*) and AT-binding transcription factor 1 (*ATBF1*) were further investigated given their functions as potential TSGs. *NQO1 *has been implicated in carcinogenesis due to its role in quinone detoxification and in stabilization of p53. One inactive polymorphic variant of *NQO1 *encodes a product showing reduced enzymatic activity. However, we did not find preferential targeting of the active *NQO1 *allele in tumors with LOH at 16q. Immunohistochemical analysis of 354 invasive breast tumors revealed that NQO1 protein expression in a subset of breast tumors is higher than in normal epithelium, which contradicts its proposed role as a tumor suppressor gene.

*ATBF1 *has been suggested as a target for LOH at 16q in prostate cancer. We analyzed the entire coding sequence in 48 breast tumors, but did not identify somatic sequence changes. We did find several in-frame insertions and deletions, two variants of which were reported to be somatic pathogenic mutations in prostate cancer. Here, we show that these variants are also present in the germline in 2.5% of 550 breast cancer patients and 2.9% of 175 healthy controls. This indicates that the frequency of these variants is not increased in breast cancer patients. Moreover, there is no preferential LOH of the wildtype allele in breast tumors.

**Conclusion:**

Two likely candidate TSGs at 16q in breast cancer, *NQO1 *and *ATBF1*, were identified here as showing reduced expression in tumors with 16q LOH, but further analysis indicated that they are not target genes of LOH. Furthermore, our results call into question the validity of the previously reported pathogenic variants of the *ATBF1 *gene.

## Background

Chromosome arm 16q is one of the regions most frequently involved in loss of heterozygosity (LOH) in breast cancer [1]. Detailed mapping shows that at least two separate regions are targeted, implying the presence of more than one tumor suppressor gene (TSG) [2]. The *CDH1 *gene, encoding the homotypic adhesion molecule E-cadherin and located at 16q22.1, was identified as the target of LOH at 16q, but gene-truncating mutations were identified only in the relatively infrequent lobular histological subtype [3]. *CDH1 *shows no mutations in tumors with LOH at 16q of the more common ductal subtype. A few candidate tumor suppressor genes have been suggested, in particular *CTCF*, a gene located at 16q22.1 that encodes an insulator. Mutations in *CTCF *have been identified in breast cancer, but only in a very small subset of cases [4,5]. Mutations in other candidate genes at chromosome 16q could not be identified, although decreased expression has been reported for some cases, suggesting that haploinsufficiency could be a mechanism of tumorigenesis [6,7]. Thus, the major TSGs at chromosome 16q in ductal breast cancer remain to be identified [8].

Recently, the gene for AT motif-binding factor 1 (*ATBF1*) was reported as a TSG at chromosome arm 16q in prostate cancer. This designation was based on the fact that *ATBF1 *is located in the smallest region of overlap affected by loss of heterozygosity (LOH), and mutations were detected in prostate cancer cell lines, -xenografts, and -tumors [9]. *ATBF1 *encodes a transcription factor with four homeobox domains and 23 Zn-fingers and was first identified as a suppressor of alpha-fetoprotein transcription [10]. ATBF1 was shown to repress expression of the *c-Myb *oncogene [11], and it may activate the cell cycle inhibitor p21 [12].

With the goal of identifying new candidate genes for the 16q TSG, we constructed a cDNA microarray containing all known and predicted genes at chromosome arm 16q, and we used it to screen breast tumor RNA for genes that are down-regulated in tumors with LOH at 16q compared to tumors without LOH. One of the down-regulated genes is *NQO1*, which encodes NAD(P)H dehydrogenase quinone 1, a phase II 2-electron reductase that detoxifies metabolites of benzene and protects the p53 tumor suppressor protein from degradation [13,14].

In this paper, we report the identification of genes at chromosome arm 16q that are significantly down-regulated in breast tumors with LOH at chromosome 16 using a cDNA microarray enriched for genes on the long arm of this chromosome. *NQO1 *and *ATBF1 *were identified as the most promising candidate TSGs. We examined in detail a polymorphism that affects the stability of the NQO1 protein in relation to LOH at 16q, but we failed to demonstrate preferential loss of one specific allele in tumors showing LOH at 16q. We did observe an association between NQO1 protein expression and histological grade.

Furthermore, we performed mutational analysis of the entire open reading frame of the *ATBF1 *sequence in 48 breast tumors with known LOH status. We found the same types of sequence variations as previously reported for prostate cancer [9], but we observed that these variations are not tumor-specific and that the wild-type allele is not a specific target of LOH at 16q. This suggests that some of the previously reported mutations in prostate cancer may have been erroneously reported as pathogenic.

## Methods

### Patient material

RNA for cDNA microarray analysis and quantitative PCR, and DNA for sequencing and LOH analysis, was isolated from fresh frozen tumor tissue (n = 39). Prior to RNA/DNA isolation, H&E sections were evaluated by a pathologist (VTBHMS) to select tissue blocks with at least 70% tumor cells. The LOH status at chromosome arm 16q had previously been determined in detail by using polymorphic markers [2]. Characteristics of the 39 tumors used in this analysis are shown in Table 1. For all tumors, non-neoplastic DNA from peripheral blood lymphocytes of the same patients was available. Normal mammary tissue was obtained from two cosmetic mammary reduction surgery specimens. All specimens were handled according to the ethical guidelines as described in the 'Code for Proper Secondary Use of Human Tissue in the Netherlands,' by the Dutch Federation of Medical Scientific Societies.

**Table 1:** Tumors used for hybridization with a 16q specific cDNA array

**DNA_No**	**16q status**	**Grade**
BT0374	Retention	I
BT0532	Retention	II
BT0538	Retention	II
BT0642	Retention	I
BT0731	Retention	III
BT0653	Retention	I
BT0681	Retention	III
BT0763	Retention	II
BT0677	Retention	U
BT0602	Retention	II
BT0614	Retention	II
BT0498	LOH entire 16q	II
BT0605	LOH entire 16q	I
BT0621	LOH entire 16q	I
BT0654	LOH entire 16q	I
BT0673	LOH entire 16q	I
BT0735	LOH entire 16q	I
BT0768	LOH entire 16q	II
BT0631	LOH entire 16q	U
BT0578	LOH entire 16q	II
BT0597	LOH entire 16q	II
BT0573	LOH entire 16q	III
BT0598	LOH entire 16q	II
BT0604	LOH entire 16q	II
BT0540	LOH entire 16q	I
BT0563	LOH entire 16q	I
BT0260	LOH 16q2-ter	II
BT0337	LOH 16q2-ter	II
BT0509	LOH 16q2-ter	III
BT0644	LOH 16q2-ter	II
BT0753	LOH 16q2-ter	II
BT0193	LOH 16q2-ter	U
BT0413	LOH 16q2-ter	III
BT0446	LOH 16q2-ter	I
BT0559	LOH 16q24	III
BT0757	LOH 16q24	I
BT0410	LOH 16q22	I
BT0505	LOH 16q22	III
BT0335	LOH 16q22	II

U = unknown

DNA from peripheral blood of 541 breast cancer patients was obtained within the ORIGO project [15]. Healthy control DNA (n = 172) was isolated from buffy coats obtained from healthy blood donors.

### cDNA microarray analysis with a 16q-specific array

All known transcripts in the 16q21-ter region that were reported in NCBI, UCSC, Ensembl, and Celera were collected from the Research Genetics sequence verified clone collection (Invitrogen, The Netherlands), from RZPD [16], or the Kazusa DNA reagent institute [17], or PCR amplified from cDNA using gene-specific primers. For each transcript, at least one cDNA clone was collected, leading to a total of 702 clones representing approximately 450 genes. Furthermore, 2000 non-selected cDNA clones obtained from the Research Genetics sequence verified clone collection were included for normalization of the 16q gene expression data. All cDNA clones were printed on the glass slides at least four times, resulting in 18,026 spots. Microarrays were constructed, hybridized, and analyzed as described previously [18]. Tumor RNA was labelled with fluorescein and Cy3, and reference RNA with biotin and Cy5 using the Micromax TSA Labeling Kit (Perkin Elmer). The reference RNA consisted of a pool of equal amounts of RNAs from a panel of cell lines (HL-60, K562, NCI-H226, COLO205, SNB-19, LOX-IMVI, OVCAR-3, OVCAR-4, CAK-IPC-3, MCF7, Hs578T, MCF10F, MCF12A), similar to the panel previously described [19].

For the identification of differentially expressed genes, R version 1.9.0 [20] using the Limma package of Bioconductor [21] was applied as previously described [18].

### mRNA expression analysis

For all five genes that showed a significant decrease in expression in breast tumors with LOH at 16q compared to breast tumors without LOH, transcript expression was determined by quantitative reverse transcriptase PCR (qPCR) on the same RNA used for cDNA microarray analysis and quantitative RT-PCR as previously described [18,22]. Briefly, SybrGreen was used to visualize PCR product formation in real time on a Bio-Rad iCycler (Bio-Rad, Hercules, CA). The geometric mean of three housekeeping genes (*HNRPM, CPSF6*, and *TBP*) was used to normalize the expression [22,23]. Primer sequences have been submitted to the RTPrimerDB (see Availability and requirements section for URL). Correlation between the expression levels measured by cDNA microarray and RT-qPCR was determined using the statistical correlation function in Microsoft Excel.

### Sequencing of *ATBF1 *in 48 breast tumors

The entire coding sequence of the ATBF1 gene was determined by generating PCR products using 44 primer pairs, as previously described [9]. PCR fragments were sequenced on an ABI 3700 DNA Analyzer. Sequences were analyzed with Mutation Surveyor™ DNA variant analysis software version 2.61 (Softgenetics, State College, PA).

### Fragment and LOH analysis

Sequencing resulted in the detection of four polymorphic repeats, one glutamic acid stretch, and three glutamine stretches. To analyze the presence of these polymorphisms, 20 ng of DNA was PCR amplified using FAM-labelled primers with the same sequence as the primers used for DNA sequencing. The fragments were analyzed on an ABI 3130 genetic analyzer, and the peaks were analyzed using GeneMapper software version 3.7 (Applied Biosystems, Foster City, CA).

LOH at these repeats was analyzed by testing the same primers on DNA from peripheral blood and breast tumors of the same patient for those cases that were heterozygous for a repeat, and by comparing peak ratios as previously described [2]. The Student's T-test was used to calculate possible over- or underrepresentation of one of the four amino acid repeat polymorphisms in breast cancer patients versus controls.

### Immunohistochemical analysis on tissue arrays

A tissue array containing paraffin-embedded formalin fixed primary breast tumor tissues has been described previously [24]. Sections of 4 μM were mounted on glass slides using a PSA^® ^Paraffin Sectioning Kit (Alphelis, Plaisir, France), and subsequently stained according to standard immunohistochemical procedures using citrate antigen retrieval. The primary antibody recognized NQO1 (A180; Santa Cruz Biotechnology, Santa Cruz, CA), and it was used at a dilution of 1:1500. Human kidney tissue was used as a positive control. The staining pattern observed was identical to that reported previously in kidney podocytes [25]. As a negative control, slides were incubated with mouse IgG1 antibody, corresponding to the isotype of the NQO1 antibody.

Stainings were semi-quantitatively scored by two observers based on visual inspection, and grouped into one of three categories: complete absence of staining (0), weak staining (1), or strong staining (2). Statistical analysis of NQO1 staining was carried out in correlation with other clinical and histological parameters as reported previously [24]; these parameters included age, stage, grade, histology, lymph node status, status of estrogen and progesterone receptors, and expression of p53 and HER2/Neu proteins. Statistical comparisons were made using the Chi-squared test for comparisons of categorical variables and ANOVA for continuous variables. All analyses were performed using SPSS version 12.01 (SPSS Inc, Chicago, IL). A Bonferroni correction for significance was applied by dividing the standard significant p-value (0.05) by the number of different conditions analyzed (n = 9), resulting in a significance cut-off of p < 0.0055.

## Results

### Differentially expressed genes in tumors with LOH at 16q detected by cDNA microarray analysis

A cDNA microarray was constructed to be highly enriched for clones representing 450 known and putative genes from 16q21-16qter which is the region most frequently affected by LOH. The cDNA microarray contained 702 chromosome 16q specific cDNA clones and 2000 cDNAs from the entire genome printed in quadruplicate. The array was hybridized with RNA from 39 breast tumors for which extensive LOH analysis at 16q had been previously performed [2]. All tumors were of the ductal subtype, and their grade and LOH status at 16q are listed in Table 1. Raw data obtained from the array hybridizations, including the list of clones on the micorarray, can be found as additional file 1 in ref. [26].

**Figure 1:**
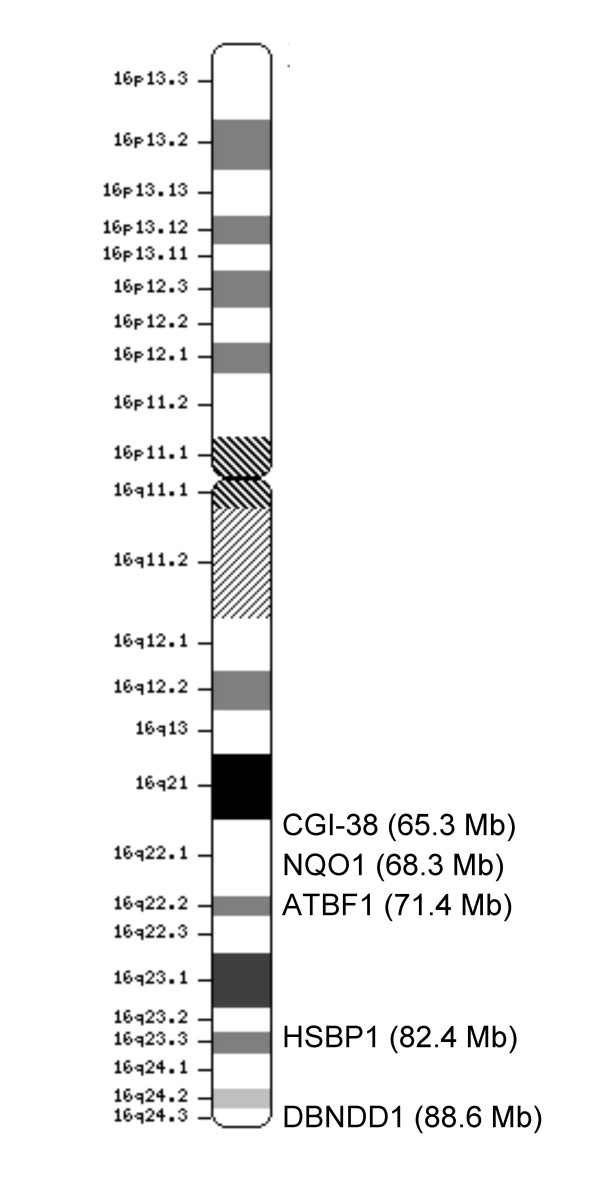
Map of chromosome 16 showing the location of genes down-regulated in breast tumors with LOH at 16q.

After normalization of the data, a Limma analysis was performed to identify genes with differential expression when comparing tumors with and without LOH at chromosome 16q. The Limma (linear models for microarray data) package of Bioconductor [21] is a moderated T-statistic that detects differentially expressed genes between groups by taking into account the natural variance within these groups and correcting for false discovery due to multiple testing. The following genes on chromosome arm 16q were expressed at significantly lower levels (p < 0.05) in tumors with LOH at 16q compared to tumors without LOH: NAD(P)H dehydrogenase quinone 1 (*NQO1*), AT-binding transcription factor 1 (*ATBF1*), dysbindin (*DBNDD1*), heat shock factor binding protein 1 (*HSBP1*), and a brain-specific hypothetical protein (*CGI-38*). Fig. 1 shows a map of chromosome 16q and the location of these five genes, with their coordinates as determined on the UCSC Genome Browser (see Availability and requirements for URL).

**Figure 2:**
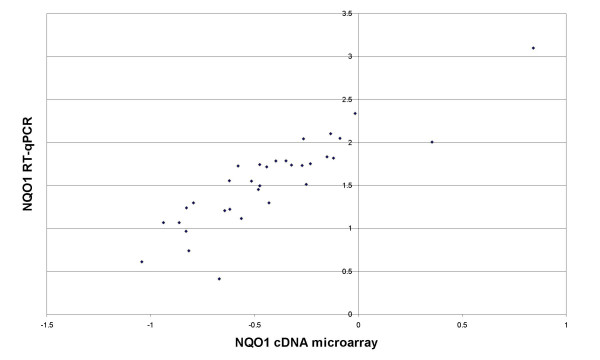
**mRNA expression of *NQO1***. Comparison of *NQO1 *mRNA expression data assessed bycDNA microarray (y-axis) and quantitative reverse transcriptase PCR (x-axis). Microarray data are shown as log_10_-transformed, normalized ratios; qPCR data are shown as the log_10_-transformed, normalized starting quantities of the mRNA.

In order to confirm the cDNA microarray data, quantitative reverse transcriptase PCR (qPCR) was performed on cDNAs from the same samples that were tested on the array. Fig. 2 shows the qPCR and microarray data for *NQO1 *plotted against each other; the two approaches show strong correlation of 88%. Correlation for the other genes was 16% for *ATBF1*, 80% for *HSBP1*, 79% for *DNBDD1 *and, surprisingly, a negative correlation of -31% for *CGI-38*. We also tested whether the expression level of *CDH1*, the gene encoding E-cadherin, differed significantly between tumors with and without LOH at 16q, but the was not significant. The correlation between RT-qPCR and microarray for *CDH1 *was quite good (88%).

*NQO1 *and *ATBF1 *have previously been reported to be involved in carcinogenesis [9,27], and were therefore investigated in more depth in breast tumors in this study.

### The C609T polymorphism in *NQO1*

A polymorphism in *NQO1 *resulting in the amino acid substitution Pro187Ser has previously been reported to reduce quinone reductase activity *in vitro *[28]. We therefore investigated the occurrence of this polymorphism in 178 patients with sporadic breast cancer. The results show that there is no prevalence of the inactive T-allele in this cohort, since the distribution of C and T (78% and 22%, respectively) did not differ from the normal distribution (Table 2) [14]. We hypothesized that if *NQO1 *is a candidate target for LOH at 16q, there should be preferential loss of the active C-allele in breast cancer. In total, of 60 cases heterozygous for the C609T polymorphism 30% (n = 18) showed retention of both alleles. Of the remaining 42 cases with LOH, 64% (n = 27) showed LOH of the T-allele and only 36% (n = 15) showed LOH of the active allele, which contradicts the hypothesis that LOH at 16q may be directed at the loss of an active allele of *NQO1*. The prevalence for LOH at the inactive allele was not significant (p = 0.06).

**Table 2:** NQO1 polymorphism C609T in breast cancer patients

Genotype	Nr of breast cancer patients	LOH
C/C	109	
		Retention: 18 (30%)
C/T	60	Loss of C-allele: 15 (25%)
		Loss of T-allele: 27 (45%)
T/T	9	

Finally, we assessed the 16q LOH status of a subset of cases using polymorphic microsatellite markers as previously described [8]. Analyzing 30 cases with *NQO1 *LOH revealed that also other markers on chromosome arm 16q show LOH. Remarkably, five cases with 16q retention showed specific LOH of NQO1: two of these cases showed LOH of the T-allele, while three showed LOH of the C-allele.

### Tissue array analysis of NQO1 protein expression in breast cancer

**Figure 3:**
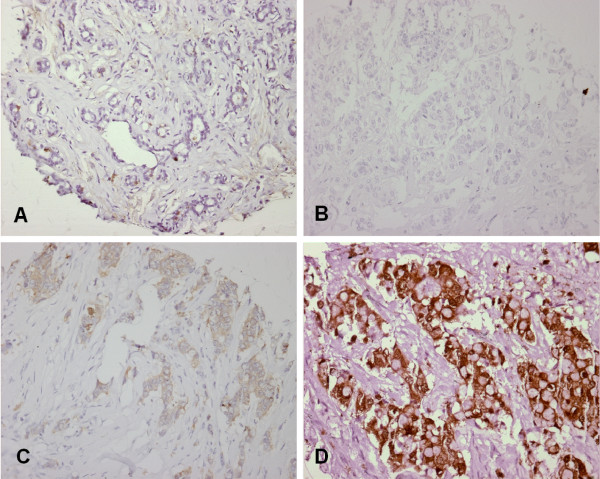
**Immunohistochemical staining for NQO1**. A, normal, negative epithelium; B, negative tumor (0); C, weakly positive tumor (1); D, positive tumor (2).

Protein expression of NQO1 was studied by immunohistochemistry on tissue arrays of invasive breast tumor tissue. In total 354 tumors were successfully examined. Examples of the staining are shown in Fig 3. Normal breast epithelium showed no or very weak cytoplasmic NQO1 staining. In tumors cytoplasmic staining of tumors varied from entirely absent to strong. A summary of the results is given in Table 3. Statistical analysis identified a significant difference (p = 0.005) in NQO1 expression between grade 1 tumors compared to grade 2 or 3 tumors, with grade 1 tumors more often showing no or weak expression (Table 3), similar to normal breast epithelium. Furthermore, strong NQO1 staining was significantly associated with positive HER2/Neu protein expression and positive progesterone receptor (PR) staining. The association with PR expression, however, was not significant when a Bonferroni correction was applied to the p-value. Other clinical and histological markers were not associated with NQO1 staining. The patient cohort used to create the tissue array overlapped minimally with the cohort for whom the LOH at 16q had been determined. The status of 16q LOH was known for 13 cases, with nine showing LOH and four showing retention. However, there was no significant association between *NQO1 *status and LOH at 16q.

**Table 3:** NQO1 immunohistochemical results

**NQO1 score**	**nr cases**		
0 (negative)	152 (43%)		
1 (weak)	132 (37%)		
2 (strong)	70 (20%)		
total	354		
	**Grade 1**	**Grade 2 or 3**	**p-value**
0 or 1	97 (35%)	179 (65%)	0.005
2	12 (18%)	56 (82%)	
	**HER2-negative**	**HER2-positive**	
0 or 1	218 (82%)	48 (18%)	0.005
2	43 (66.2%)	22 (34%)	
	**PR-negative**	**PR-positive**	
0 or 1	134 (49%)	137 (51%)	0.024*
2	44 (65%)	24 (35%)	

* not significant after Bonferroni correction

### DNA sequencing of the entire *ATBF1 *coding sequence in 48 breast tumors with known chromosome 16q LOH status

In order to identify somatic mutations in *ATBF1 *similar to those reported for prostate cancer [9], we sequenced the entire coding sequence of the gene using PCR primers as previously described [9]. The *ATBF1 *coding sequence from 43 primary breast tumor samples, selected to contain at least 50% tumor cells, was PCR amplified and sequenced using the forward PCR primers. This set of tumors was enriched for cases with LOH at 16q, only five cases of which showed 16q retention. Possible sequence variations were confirmed by sequencing the opposite strand. The results of all variants for which the sequencing was consistent in both directions are listed in Table 4. Most of the variations could be identified as polymorphisms, since they were reported as such in either the ENSEMBL database [29] or by Sun et al. [9]. We detected five novel single base pair variations that have not previously been reported, and which therefore may be pathogenic mutations, however, the same base pair variation was always detected in germline DNA isolated from non-neoplastic peripheral blood cells, so they are excluded as somatic mutations.

**Table 4:** Variants identified in ATBF1 in 48 breast cancer samples

**Nucleotide change**	**Aminoacid change**	**Polymorphism because**
*Synonymous polymorphisms*		
1012C>T	113Ala	Found multiple times (7/48)
1096A>G	141Ala	Reported in Ensembl as polymorphism
2443G>A	590Phe	Reported in Ensembl as polymorphism
2497T>C	608Ser	Reported by Sun et al. as polymorphism
3058C>G	795Pro	Reported in Ensembl as polymorphism
3340C>T	889Ser	Reported in Ensembl as polymorphism
3589G>A	972Ser	Reported in Ensembl as polymorphism
4018C>T	1115Ser	Also present in normal matched sample
4297G>T	1208Ser	Also present in normal matched sample
4756C>T	1361Ile	Reported in Ensembl as polymorphism
5119A>T	1482Ala	Reported in Ensembl as polymorphism
6292C>T	1873His	Reported in Ensembl as polymorphism
6829G>A	2052Pro	Also present in normal matched sample
7714A>G	2347Gln	Reported in Ensembl as polymorphism
7828G>A	2385Pro	Reported by Sun et al. as polymorphism
8851T>C	2726Leu	Reported in Ensembl as polymorphism
9496A>G	2941Gly	Reported in Ensembl as polymorphism
10552C>T	3293Ala	Reported in Ensembl as polymorphism
*Non-synonymous polymorphism*		
858C>T	Ala62Val	Also present in normal matched sample
887A>C	Ser72Ala	Reported in Ensembl as polymorphism
2053C>G	Glu460Gln	Reported in Ensembl as polymorphism
2304C>T	Ser544Leu	Also present in normal matched sample
3003A>G	Val777Ala	Reported in Ensembl as polymorphism
3515G>A	Ala948Ile	Also present in normal matched sample
3662G>T	Ala997Ser	Reported in Ensembl as polymorphism
6143C>G	Leu1824Val	Reported by Sun et al. as polymorphism
6715G>C	Gln2014His	Reported by Sun et al. as polymorphism

Remarkably, we also identified insertions and deletions in four different amino acid repeats present in this gene (Table 5). The indels were all in-frame and resulted in a shorter or longer glutamic acid or glutamine amino acid repeat. Two of these variants, located in exons 9 and 10, were reported by Sun et al [9] as somatic pathogenic mutations in two and nine tumor samples, respectively. We tested all four insertions/deletions in the corresponding normal DNA, isolated from peripheral blood, and found that all variants were also present in this non-neoplastic tissue. This clearly shows that these variants are not somatic mutations in breast cancer.

**Table 5:** Aminoacid repeat length variations in ATBF1

**Exon nr**	**AA position**	**AA Repeat**	**Nature – frequency**	In tumors*	**Allele loss**	**In normals**	In BC patients**
2	487	Glu	15 bp del – 1	4/116 (3.4%)	1 wt	1/172 (0.6%)	7/541 (1.3%)
			12 bp ins – 11		2 variants 1 no loss		
9	1741	Gln	3 bp del – 1	2/110 (1.8%)	2 wt	5/162 (3.1%)	11/539 (2.0%)
			6 bp del – 4				
			3 bp ins – 4				
			6 bp ins – 10				
10	3202	Gln	21 bp del – 8	1/98 (1%)	1 variant	1/146 (0.7%)	6/530 (1.1%)
10	3380	Gln	3 bp del – 5	6/115 (5.2%)	2 wt	11/148 (7.4%)	27/351 (7.7%)
			6 bp del – 15		4 Variants		
			3 bp ins – 24				

* All normal DNAs of these patients show the same variant.

** Tested on normal DNA from peripheral blood cells

Alternatively, a possible pathogenic nature of the four amino acid repeat polymorphisms in breast cancer might be indicated by the detection of preferential loss of the wildtype allele in tumors. The results are summarized in Table 5. In total, we identified amino acid polymorphisms in 13 out of 116 breast tumor patients, which were selected to show LOH at 16q. In five cases, the wildtype allele was affected by LOH, whereas in the other eight cases it was retained and either the variant allele was lost (n = 6) or there was no LOH (n = 1). This latter case, in fact, showed LOH only at the most distal part of 16q, a region that does not contain the *ATBF1 *gene. The amino acid length polymorphism in exon 9 was found in only two samples, and the wild-type allele was lost in both cases. The other three polymorphisms showed LOH of both the wildtype and variant alleles.

Furthermore, we tested whether one or more of these variants could confer increased risk of breast cancer by comparing their frequency in a population of 550 breast tumor patients with their frequency in a cohort of 175 healthy controls from the same geographical location. In total, a variant allele for one of the four polymorphisms was detected 18 times in 628 successful PCR reactions (2.9%) in normal controls, whereas a variant allele was found 49 times in 1961 reactions (2.5%) in the germline DNA of breast cancer patients. In addition, no significant difference could be found between the cases and controls for any of the four separate variants (Table 5).

## Discussion

The frequent occurrence of loss of heterozygosity (LOH) at the long arm of chromosome 16 in breast cancer has led many investigators to search for the tumor suppressor genes that may be affected by LOH in this chromosomal region. Many genes have been proposed as putative candidates. In particular, *FBXO31 *was recently identified not by deletion mapping, but by identification of its ability to induce senescence in tumor cells [7]. However, none of these genes follow the classical two-hit model of Knudson [30]. In an attempt to assess all known genes at chromosome 16q simultaneously within a single experiment, we designed a chromosome 16q-specific cDNA microarray and hybridized the array with RNAs from a series of well-characterized breast tumors. By comparing tumors with and without LOH at the 16q arm, we identified a small set of genes that met our strict statistical standards.

In the present work, we were unable to stratify the groups based on histological grade or the smallest region of overlap involved in LOH, since larger cohorts of patients are needed for this analysis.

Two genes that showed lower mRNA expression in breast tumors with LOH at chromosome arm 16q were of particular interest for breast cancer and were therefore subjected to more detailed investigation. The most significantly down-regulated gene was *NQO1 *encoding NAD(P)H dehydrogenase quinone 1. This gene has been implicated in carcinogenesis because of its role in the reduction and detoxification of quinones and their derivatives, thereby protecting cells from oxidative damage [14]. Moreover, NQO1 has been shown to stabilize the p53 tumor suppressor protein by inhibiting its degradation through a direct protein-protein interaction [13,31]. The possible role of *NQO1 *in carcinogenesis has been studied extensively, especially the occurrence of a frequent polymorphism, 609C>T, which results in a proline to serine substitution at amino acid 187 [32]. This substitution results in a variant with poor enzymatic activity and no detectable protein, as shown in individuals homozygous for the T-allele [33]. The prevalence of the 609T allele of *NQO1 *has been studied in tumors of lung, bladder, and colorectal cancer as well as leukaemia. However, the results are inconsistent [14] and no correlation between *NQO1 *allele frequency and breast cancer has been observed [32,34]. This is consistent with our findings. To our knowledge there are no studies examining the preferential loss of heterozygosity of the active 609C allele. A possible role for *NQO1 *in breast cancer would be confirmed if it could be shown that LOH at 16q is targeted at the active allele. However, we could not show a prevalence of 609C loss and, in fact, LOH was higher at the 609T allele. It is therefore unlikely that *NQO1 *is the target tumor suppressor gene at 16q. Nevertheless, the high incidence of LOH (70%) at this locus suggests that the gene lies near another target gene.

We also investigated NQO1 protein expression in 354 breast tumors. Normal breast epithelium showed no or only weak expression of this protein, whereas 20% of breast tumors showed strong staining. This is not consistent with a tumor suppressor function for *NQO1*. Remarkably, intermediate and poorly differentiated tumors (grade 2 and 3) showed overexpression of NQO1 protein more often than those showing low differentiation (grade 1). We have previously shown that grade 1 tumors have LOH at 16q through physical loss, whereas poorly differentiated breast tumors show 16q LOH through mitotic recombination [35]. Therefore, the absence of NQO1 expression may be attributed to physical loss of 16q and LOH at 16q may not be specifically targeted at the *NQO1 *gene. In addition, the expression of *HER2 *and *NQO1 *showed significant correlation. Like *NQO1*, also *HER2 *overexpression was more prevalent in poorly differentiated breast tumors, which could explain this correlation.

*ATBF1 *was an other candidate gene identified by our microarray analysis showing decreased mRNA expression in tumors with LOH at 16q. This gene was recently reported to contain somatic mutations in prostate cancer, a tumor type in which frequent LOH at 16q is also a hallmark [9]. ATBF1 is one of the largest transcription factors known, containing four homeodomains and 23 zinc fingers [10]. Nevertheless, little is known about its function. The ATBF1 protein binds to the AT motif of the alpha-fetoprotein (*AFP*) gene, thereby inhibiting its transcription. A possible role in tumorigenesis has been found in AFP-producing gastric cancer, an aggressive tumor type that lacks *ATBF1 *[36]. Interestingly, ATBF1 was found to bind to Myb oncoprotein, as well as to inhibit transcription of the *MYB *gene [11]. Combined with the identification of somatic mutations in prostate cancer [9] these features are consistent with a tumor suppressor function for *ATBF1*. It has been suggested that in breast cancer, higher mRNA expression of *ATBF1 *is associated with a better prognosis, i.e. absence of tumor-positive lymph nodes [37]. In a previous study of 712 breast tumors we analyzed a possible association between positive lymph nodes and LOH status at 16q [8], however, we failed to demonstrate a significant correlation. Since a decrease in mRNA is associated with 16q LOH, but lymph node status is not, our current study cannot support the observation by Zhang et al. that ATBF1 expression is correlated with better prognosis in breast cancer [37].

Screening of the entire *ATBF1 *open reading frame of 43 breast tumor DNAs by direct sequencing revealed no somatic mutations, only previously described single nucleotide polymorphisms (SNP) and 7 seven new SNPs. All of these SNPs were also present in matched normal DNA. Although we cannot exclude that these variants are pathogenic, this seems quite unlikely because linkage of chromosome arm 16q and hereditary breast cancer has never been demonstrated [38].

We confirmed the same in-frame deletions in contiguous stretches of glutamic acid codons in exons 9 and 10 as described by Sun et al. [9] in prostate cancer. However, whereas those authors reported that these mutations were somatic, we have shown here that they were also present in matched normal DNA from peripheral blood cells, confirming these variants as germline. In addition we identified similar germline variations at two different locations in the gene that affect the amino acid repeat length of stretches of glutamine and glutamic acid residues. As in the case of the *NQO1 *polymorphism we could not show neither preferential LOH of these repeats nor a possible role in predisposing towards breast carcinogenesis. According to our results these repeat length variations should be considered as polymorphic variations. The effect of these variations should be further investigated using functional assays. A dramatic effect is not very likely given the fact that the variations are bidirectional: both insertions and deletions were observed in three of four variants. Recently Xu and co-workers [39] reported a 21- or 24-nucleotide deletion at position 3381 in *ATBF1 *in the germline of prostate cancer patients and concluded that these variants are associated with prostate cancer risk. We also found deletions at the same glutamine tract, but they were only three or six base pairs in length and they showed no significant difference in frequency between breast cancer patients and normal controls.

The large number of variations found in *ATBF1 *can be explained by the size of the gene, which is exceptionally large, with over 11 kb of coding sequence. The presence of simple sequence repeats coding for amino acid repeats ranging from 8 to 26 residues may explain the high frequency of length variations. Repeat length variations can lead to pathogenic traits, often neurological in character. However, these variations are almost invariably expansions that increase over time [40]. All repeats detected in *ATBF1 *showed the same length in tumor and normal DNA, and were in some cases even shorter than the most frequent allele (Table 3). A recently published study described the sequencing of the *ATBF1 *open reading frame in 32 breast cancer cell lines [41]. Two possible mutations were identified, one as an amino acid substitution at codon 2622 and one undefined change in a poly(T) tract. Whether these mutations were pathogenic was not verified.

Three other genes were identified to have a significantly lower expression in breast tumors with LOH at chromosome 16q compared to tumors without LOH. The difference in expression level for one of these, *CGI-38*, a brain-specific transcript of unknown function, could not be confirmed by RT-qPCR. Expression levels of the other two, dysbindin domain containing 1 (DBNDD1) and heat shock binding protein 1 (HSBP1) could be confirmed by RT-qPCR and may be the subject of future breast cancer research.

## Conclusion

Using a chromosome 16q-specific cDNA array to identify tumor suppressor genes we have found five candidates, of which the two most interesting, *NQO1 *and *ATBF1*, were excluded as classical candidate genes. Of special note are our results indicating that *ATBF1 *variants showing differences in the length of amino acid repeat sequences are most likely non-pathogenic and instead are normally occurring polymorphisms. This calls into question the conclusions of Sun et al. [9], who found these variants to be somatic mutations involved in prostate cancer.

New views on the role of loss of heterozygosity in cancer are currently of importance for their relevance to the targeting of multiple genes [42,43] and haploinsuffiency [44]. These views promise to provide better working hypotheses for elucidating somatic cancer genetics than Knudson's two-hit model.

## Competing interests

The author(s) declare that they have no competing interests.

## Availability and requirements

Primer sequences have been submitted to the RTPrimerDB: 

UCSC Genome Browser: 

## Authors' contributions

AMCJ conceived, designed, and coordinated the study, as well as drafted the manuscript. RVE performed the sequence analysis of *ATBF1*. ML assisted in the construction of the cDNA microarray and RNA isolation, and analyzed the mRNA expression data. MKS designed the tissue array and performed the statistical analysis. LVVeer conceived and coordinated the tissue array. KP performed the qRT-PCRs and the *NQO1 *genetic analyses. RZ assisted in the hybridization and analysis of the cDNA microarray. JLP reviewed the tissue sections for constructing the tissue array. VTHBMS reviewed the breast cancer cases for the expression and genetic analyses. TVW designed the cDNA microarray, coordinated the mRNA expression studies, and helped draft the manuscript. CJC is the principal investigator of the work described and helped in conceiving the study and drafting the manuscript.

## Pre-publication history

The pre-publication history for this paper can be accessed here:


